# Technology in the management of diabetes in hospitalised adults

**DOI:** 10.1007/s00125-024-06206-4

**Published:** 2024-07-02

**Authors:** Hood Thabit, Jonathan Schofield

**Affiliations:** 1 Diabetes, Endocrinology and Metabolism Centre, Manchester Royal Infirmary, Manchester University NHS Foundation Trust, Manchester, UK; 2 Division of Diabetes, Endocrinology and Gastroenterology, School of Medical Sciences, Faculty of Biology, Medicine and Health, University of Manchester, Manchester, UK

**Keywords:** Automated insulin delivery, Clinical decision support, Diabetes technology, Glucose sensor, Hospital, Non-critical care, Review

## Abstract

Suboptimal glycaemic management in hospitals has been associated with adverse clinical outcomes and increased financial costs to healthcare systems. Despite the availability of guidelines for inpatient glycaemic management, implementation remains challenging because of the increasing workload of clinical staff and rising prevalence of diabetes. The development of novel and innovative technologies that support the clinical workflow and address the unmet need for effective and safe inpatient diabetes care delivery is still needed. There is robust evidence that the use of diabetes technology such as continuous glucose monitoring and closed-loop insulin delivery can improve glycaemic management in outpatient settings; however, relatively little is known of its potential benefits and application in inpatient diabetes management. Emerging data from clinical studies show that diabetes technologies such as integrated clinical decision support systems can potentially mediate safer and more efficient inpatient diabetes care, while continuous glucose sensors and closed-loop systems show early promise in improving inpatient glycaemic management. This review aims to provide an overview of current evidence related to diabetes technology use in non-critical care adult inpatient settings. We highlight existing barriers that may hinder or delay implementation, as well as strategies and opportunities to facilitate the clinical readiness of inpatient diabetes technology in the future.

**Graphical Abstract:**

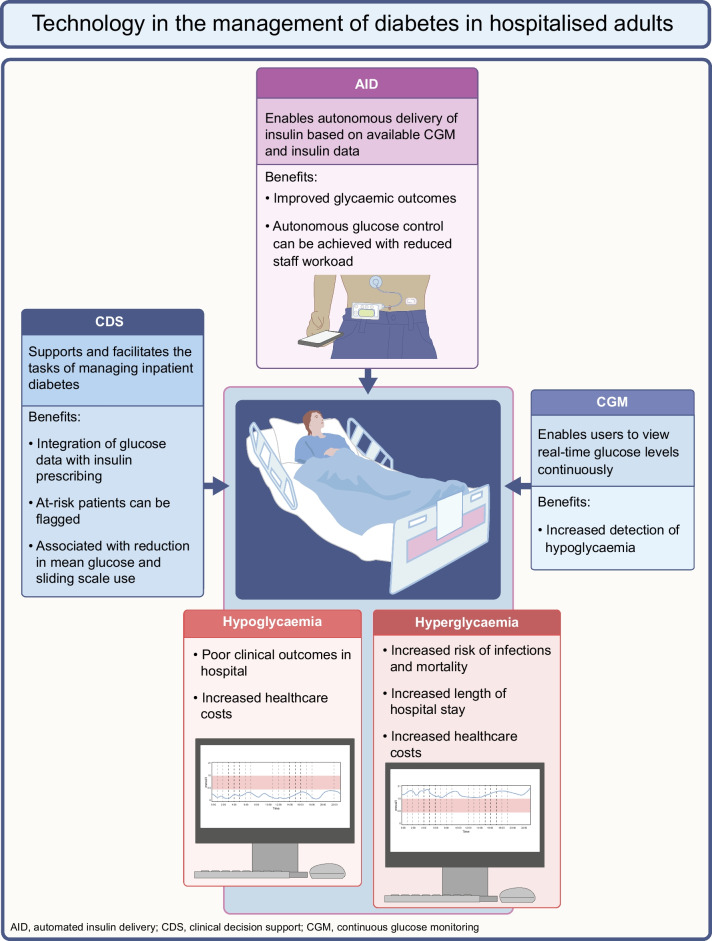

## Introduction

The number of adults worldwide living with diabetes has been rising over the past decade [1]. This is reflected in the increased prevalence of inpatient diabetes. It is estimated that approximately 25–30% of adults in hospital have diabetes [2] and that over 30% of inpatients with hyperglycaemia were previously undiagnosed with diabetes [3]. Hyperglycaemia in hospitalised patients has been associated with adverse clinical outcomes such as increased risk of infections and mortality and increased length of stay (LOS), with subsequent increased financial costs to healthcare systems [4–6]. Interventions to optimise glycaemic management in non-critical care settings have been shown to be associated with a 60% reduction in the risk of infection [7]. Similarly, episodes of hypoglycaemia in hospital are associated with poor outcomes and increased costs, which might also reflect the severity of illness and higher rates of comorbidities in these inpatients [8]. In the USA, over 40% of the total expenditure for diabetes in 2012 (US$245 billion) was related to hospital admissions [9]. Other international data have also consistently shown that inpatient diabetes and its complications are associated with high healthcare expenditure and prolonged LOS [10–12].

Despite the availability of national and international guidelines on managing inpatient diabetes, optimising glycaemic management in hospital remains challenging for reasons that will be discussed below. The use of medical devices and healthcare technology has been gaining traction over the past decade, playing important roles such as supporting therapeutic management and shared decision making between people with diabetes and healthcare professionals [13–15]. The use of diabetes technology such as continuous glucose monitoring (CGM) and automated insulin delivery (AID) systems in outpatient settings is growing. Since the COVID-19 pandemic, the potential for innovation and technology transfer from ambulatory outpatient settings to inpatient diabetes has also been evolving.

The aim of this article is to provide an overview of technology application in adult inpatient diabetes management, specifically in non-critical care settings, and its potential outlook. We review current guidelines on adult inpatient diabetes management in non-critical care settings and evidence from clinical studies evaluating the feasibility and effectiveness of inpatient diabetes technology. The clinical readiness of such technologies and challenges to be addressed are also discussed. Further reviews of inpatient and critical care diabetes management are available elsewhere [16–18]. An electronic search of MEDLINE (via PubMed) and a public register of clinical trials (www.clinicaltrials.org) was conducted using keywords (‘inpatient hyperglycaemia’, ‘inpatient diabetes’, ‘non-critical care’, ‘critical care’, ‘intensive care’, ‘general wards’, ‘insulin therapy’, ‘glucose monitoring’, ‘computerised provider order entry’, ‘clinical decision support system’, ‘closed-loop system’, ‘automated insulin delivery’) combined with relevant MeSH terms. A secondary search was conducted using these keywords and terms in the abstract databases from scientific sessions, and the bibliographies of retrieved papers were searched. Additional papers known to the authors were also used.

## Current status of inpatient diabetes management

Findings from studies and national audits have revealed that inpatient diabetes management remains suboptimal, with a significant number of people with diabetes experiencing harm or prolonged episodes of hyperglycaemia in hospital as a consequence [18–20]. These individuals are usually managed by non-diabetes specialist healthcare professionals. Hyperglycaemia is also encountered in inpatients with stress hyperglycaemia and those previously undiagnosed with diabetes. Iatrogenic hypoglycaemia also presents a significant challenge to inpatient staff, as episodes tend to occur in those who are most vulnerable to the adverse consequences of hypoglycaemia, such as the elderly and those who are critically unwell. To support healthcare professionals, guidelines for inpatient management have been published by various professional societies.

### Guidelines and evidence on inpatient diabetes care in non-critical care

Good-quality evidence on inpatient diabetes management remains sparse. The majority of guidelines are therefore consensus based, which at times may be controversial and contradictory. Most evidence is derived from data collected during observational or retrospective studies, which are potentially exposed to bias and confounders [7]. A prospective RCT evaluating glycaemic management in hospital settings has been conducted but was performed in large academic and tertiary hospitals [21] and the methods and findings may not be generalisable to other hospital settings. A meta-analysis of RCTs and observational studies reported that intensive glycaemic management in the general ward was associated with a 59% reduction in the risk of infections, predominantly among post-cardiac and general surgery inpatients [7]. However, in those whose glycaemic goal was achieved during the studies, the overall risk of hypoglycaemia was increased by 58%. Thus, current guidelines are focused on minimising the risk of iatrogenic hypoglycaemia and advocate appropriate and safe glycaemic management. This is relevant as most professional societies recommend insulin therapy, which carries an inherent risk of hypoglycaemia, as the cornerstone of glycaemic management in hospitalised individuals.

Guidance from the ADA recommends initiating insulin therapy for the treatment of persistent hyperglycaemia ≥10.0 mmol/l [18], with a treatment target glucose level of 7.8–10.0 mmol/l for most critically ill and non-critically ill inpatients, but more stringent targets (6.1–7.8 mmol/l or 5.6–10.0 mmol/l) if they can be achieved without a significant hypoglycaemia burden. The Endocrine Society Clinical Practice Guideline recommends maintaining glucose levels in the range 5.6–10.0 mmol/l in adults with diabetes who are hospitalised for non-critical care illness [22]. Basal alone or basal plus bolus correction insulin regimens are preferred for those with variable or poor oral intake, while basal–bolus insulin regimens with correction components modified for nutritional status are preferred in non-critically ill individuals.

However, there are some differences between guidelines, particularly in relation to the continuation or use of non-insulin glucose lowering therapies in hospital. The ADA has suggested that further research is needed before using non-insulin glucose lowering therapies in hospital, while UK Joint British Diabetes Societies (JBDS) guidance states that usual diabetes medications, including non-insulin therapy, can be continued unless there are specific contraindications or safety concerns [23, 24]. Those with adequate oral intake are encouraged to continue their usual insulin therapy, although adjustments to insulin doses with support from the clinical team may be required. In certain cases insulin regimens may need to be changed, for example from premixed to basal–bolus insulin because of an increased risk of hypoglycaemia [25]. The JBDS has also published several healthcare professional guidelines related to the management of inpatients experiencing hyper- or hypoglycaemia, as well as inpatients receiving steroids or enteral feeding or undergoing surgery [26–28]. Information to support the self-management of diabetes in hospital is also available, as people with diabetes often have more knowledge of their own insulin and other specific requirements than the healthcare team managing them in hospital [29]. This is pertinent given that inpatient staff may also be unfamiliar with current diabetes technologies being used by people living with diabetes, many of whom will continue using these during their hospitalisation if able [30, 31].

### Implementation challenges

Strategies to improve education among healthcare professionals include ensuring safer and more effective use of insulin therapy. These have been outlined by key organisations responsible for improving the quality and safety of inpatient diabetes care [20, 32]. However, despite the available guidelines and strategies, inpatient glycaemic management remains suboptimal, with notable numbers still experiencing hypoglycaemia in hospital [33, 34]. This is likely attributable to multiple factors including the challenge of overcoming clinical inertia [35], especially when the majority of inpatients with diabetes are admitted to hospital for other conditions and are commonly under the care of healthcare professionals who may lack the required expertise in diabetes management [36]. The practice of using sliding scale insulin, which relies on corrective doses of insulin after hyperglycaemia, has occurred and is discouraged by professional societies because of the inherent risk of ‘insulin stacking’ and suboptimal glycaemic management [37, 38], but worryingly remains prevalent in the authors’ experience and published audits [39].

Compounding this challenge is the increasing number of inpatients who require regular monitoring of blood glucose levels, insulin dose adjustment and alignment of the timing of insulin intake with meals, contributing to the already significant workload of often limited numbers of ward staff [33, 40]. A national survey in the UK revealed that current inpatient diabetes staffing is inadequate to provide optimal care for people with diabetes in hospital [41]. This has led to gaps in best practice with inadequate insulin therapy management and limited glucose monitoring, which themselves increase the risk of preventable medical errors. There is therefore a growing realisation that, unless strategies to manage inpatient glycaemic management can be implemented consistently and effectively, the objectives recommended by current guidelines will continue to be unmet.

## Latest clinical evidence of diabetes technology use in hospital

Innovations in technology over the past decade have driven improvements in outpatient diabetes care [42, 43]. These range from advanced insulin bolus calculators, CGM sensors, continuous s.c. insulin infusion therapy and, more recently, AID systems. Adopting diabetes technology in hospital may potentially benefit individuals with diabetes and overcome the unmet need for better inpatient glycaemic management and alleviate the workload burden of clinical staff. The current evidence on and status of technology use in hospital settings are outlined in the following sections.

### Clinical decision support systems

Despite the availability and dissemination of professional guidelines for inpatient diabetes management, adherence remains limited [44, 45]. This may be partly attributed to the complexity of treatment algorithms and insulin protocols and fear of hypoglycaemia, especially among general ward staff who are not specialists in diabetes and are often unfamiliar with these guidelines [46]. A potential solution to reduce complexity and facilitate adherence is the use of medical software such as clinical decision support (CDS) systems designed to support and facilitate the tasks of managing inpatient diabetes. The use of CDS systems has been growing in healthcare; however, in diabetes the evidence on use of CDS systems has mostly been derived from outpatient settings [47–49]. Inpatient CDS systems are being designed to integrate with electronic medical records (EMRs) and thus reduce scenarios of severe hypoglycaemia and recurrent hyperglycaemia, while minimising inappropriate use of insulin therapy that might lead to harm [50].

There remains a lack of high-quality RCTs to support the benefits of CDS application in inpatient settings. Most evidence comes from observational and descriptive studies associating CDS use with improved glycaemic management and reduced LOS in hospital (Table 1). Researchers in Austria tested a CDS system (GlucoTab) against standard management in a non-randomised ward-controlled study involving 74 participants with type 2 diabetes [51]. The GlucoTab CDS system was used on a tablet device and was not integrated with the hospital’s EMR system. However, it provided automated workflow support including a display of blood glucose measurements and an in-built algorithm to calculate insulin doses that ward staff could administer. Compared with the control group, those using GlucoTab had a higher percentage time in range (blood glucose 3.9–10.0 mmol/l; 73% vs 53%, *p*<0.001), with high staff adherence to the algorithm-calculated insulin doses (>90%). However, more time with blood glucose <3.9 mmol/l was observed in the afternoon period in the GlucoTab group, which the authors attributed to the corrective boluses given. A larger (*n*=150) observational study of participants with type 2 diabetes on general hospital wards on multiple daily insulin injections showed that use of the GlucoTab CDS system was associated with a mean glucose level of 8.8 ± 1.8 mmol/l, with time in range (3.9–10.0 mmol/l) of 68.8% [52]. Adherence to suggested insulin doses was also high in this study at >93%, while the time spent with blood glucose <3.9 mmol/l was low at 2.2%.

**Table 1:** Overview and summary of results of selected inpatient (non-critical care) technology studies

Study	Study design	No. of participants	Intervention	Control	Main outcome/finding	Other relevant findings
Mader [51]	Non-randomised prospective study	74	GlucoTab CDS software	Usual care	GlucoTab vs control, % time in range (BG 3.9–10.0 mmol/l): 73% vs 53%, *p*<0.001	Higher % time below range (BG <3.9 mmol/l) in GlucoTab group vs control group
Pichardo-Lowden [53]	Interruptive time series	3482	GlucAlert-CDS software activated	GlucAlert-CDS software inactivated	CDS software activated vs inactivated, mean LOS with and without glycaemic gaps in care: 87.2 vs 89.0 h, *p*=0.53	
Lichtenegger [52]	Prospective observational study	150	GlucoTab CDS	None	Mean POC-BG: 8.8 ± 1.8 mmol/l	68.8% (*n*=4879) of all BG measurements were in the range 3.9–10.0 mmol/l; 29.0% and 2.3% were >10 mmol/l and ≥16.7 mmol/l, respectively; 2.2% and 0.2% were <3.9 mmo/l and <3.0 mmol/l respectively
Dillmann [96]	Prospective non-randomised pilot study (start vs end of hospitalisation)	53	RT-CGM	None	No significant differences in % time in range (3.9–10.0 mmol/l), time <3.9 mmol/l and >10.0 mmol/l BG in the whole group	In type 2 diabetes group only (*n*=25), % time in range significantly increased between the start and end of hospitalisation (*p*=0.043) and time >10.0 mmol/l BG significantly decreased (*p*=0.049). No change was observed in time <3.9 mmol/l BG
Singh [65]	RCT	72	RT-CGM	POC-BG	RT-CGM group experienced 60.4% fewer hypoglycaemic events (BG <3.9 mmol/l) than control group (0.67 events/participant [95% CI 0.34, 1.30] vs 1.69 events/participant [1.11, 2.58], *p*=0.024)	
Fortmann [64]	RCT	110	RT-CGM	POC-BG with blinded CGM (for data collection only)	Control group vs. RT-CGM group, mean (SD) glucose:13.2 (2.5) vs 12.2 (2.4) mmol/l, *p*=0.0311	Control group vs RT-CGM group, % time in range (BG 3.9–10.0 mmol/l): 19.89 (3.34–40.09) vs 25.31 (11.78–42.97), *p*=0.1460; % time in range (BG 3.9–13.9 mmol/l): 63.95 (31.25–77.95) vs 72.83 (59.03–83.57), *p*=0.0404 No significant differences between groups in time spent in hypoglycaemia (BG <3.0 mmol/l and <3.0 mmol/l)
Thabit [76]	RCT	40	Fully closed-loop AID	Conventional s.c. insulin therapy	AID vs control, % time in range (BG 5.6−10.0 mmol/l): 59.8 ± 18.7% vs 38.1 ± 16.7%, *p*=0.0004	AID vs control, % time >10.0 mmol/l BG: 30.1 ± 20.4% vs 49.1 ± 24.1%, *p*=0.011 No differences between groups were observed in % time <5.6 and <3.5 mmol/l BG
Bally [77]	Multicentre RCT	136	Fully closed-loop AID	Conventional s.c. insulin therapy	AID vs control, % time in range (BG 5.6−10.0 mmol/l): 65.8 ± 16.8% vs 41.5 ± 16.9%, *p*<0.001	AID vs control, % time >10.0 mmol/l BG: 23.6 ± 16.6% vs 49.5 ± 22.8%, *p*<0.001 No difference between groups was observed in % time <3.0 mmol/l BG
Boughton [78]	Multicentre RCT	43	Fully closed-loop AID	Conventional s.c. insulin therapy	AID vs control, % time in range (BG 5.6−10.0 mmol/l): 68.4 ± 15.5% vs 36.4 ± 26.6%, *p*<0.0001	AID vs control, % time >10.0 mmol/l BG: 22.2 ± 15.7% vs 54.8 ± 29.7%, *p*<0.0001 No differences between groups were observed in % time <3.9 and <3.0 mmol/l BG
Davis [79]	Single-arm pilot study	22	Hybrid closed-loop AID	None	% time in range (BG 3.9–10.0 mmol/l): 68 ± 16% (in *n*=16)	% time <3.9 mmol/l BG: 0.17 ± 0.3%; % time <3.0 mmol/l BG: 0.06 ± 0.2%

BG, blood glucose

There is also evidence to suggest that CDS use may be associated with reduced LOS in hospital. In an observational study performed in an academic medical centre in the USA involving 3482 participants, researchers used the GlucAlert-CDS that was integrated with the hospital EMRs and automatically detected what they defined as hospital glycaemic gaps in care (GIC) [53]. The study adopted an interrupted time series design in which the CDS tool was activated and deactivated by the researchers every 3 months over a 12 month period. Data were collected from 4788 hospital admissions. A non-significant LOS reduction was reported in all admissions with GIC (−5.7 h, *p*=0.057), while a sub-analysis showed a significant LOS reduction of 82 h in those who experienced three hypoglycaemic events during their hospitalisation. The lack of randomisation and a matched control group means that attributing causality to CDS intervention remains limited and uncertain.

The aforementioned CDS systems were designed for use with s.c. insulin therapy, specifically within non-critical care settings. Computerised insulin infusion protocol systems (CIIPs) have been developed for i.v. insulin therapy use in hospitalised individuals, such as the Glucommander CIIPs [54]. However this has mostly been used and studied in critical care settings or step-down units following admission to ICU [55–57].

### Continuous glucose monitoring

Current CGM devices measure interstitial fluid glucose concentrations every 1–5 min and enable users to view real-time glucose levels continuously; hence, they are sometimes known as real-time continuous glucose monitoring (RT-CGM) devices. These devices also provide information related to predicted glucose levels and trends, and feature hypo- and hyperglycaemia alerts that can be customised by the user as appropriate. Most CGM devices are factory calibrated and approved for non-adjuvant use in outpatient ambulatory settings, with acceptable sensor accuracy (mean absolute relative difference [MARD]) of around 8–9% [58, 59]. CGM application has been shown to improve outpatient glycaemic outcomes in various cohorts [60–62]. Despite its widespread use in ambulatory glucose management, CGM has not yet received US Food and Drug Administration (FDA) approval for use in hospitalised inpatients. The FDA, however, did not object to the use of CGM to manage hospitalised individuals during the COVID-19 pandemic, because of the perceived benefits of limiting healthcare professional and patient exposure, and several groups reported promising results from real-world use of RT-CGM within this context [63].

There remains a paucity of RCT data evaluating the effectiveness and health economic costs of CGM in inpatient settings. The largest RCT to date was performed in 110 participants with type 2 diabetes receiving insulin therapy (Table 1). The study showed that RT-CGM compared with point-of-care capillary blood glucose monitoring (POC-BG) led to a significant reduction in mean glucose levels and increased time spent in the range 3.9–13.9 mmol/l [64]. No between-group differences were found, however, in time spent in the range 3.9–10.0 mmol/l and time spent in hypoglycaemia. An RCT involving 72 insulin-treated participants with type 2 diabetes did report a reduction in hypoglycaemic events (<3.9 mmol/l) in hospital during CGM use compared with POC-BG monitoring (0.67 vs 1.69 events/participant, *p*=0.024) [65]. Both studies were conducted in large academic hospitals, thereby limiting generalisability, and data related to sensor usage and cost analysis were not reported.

An expert panel recently published a consensus definition of inpatient CGM metrics for use in research, to make it easier to compare outcomes between studies [66]. Other important considerations for CGM use in hospital are accuracy and safety, particularly in individuals who are systemically unwell and where there may be potential interference to sensor performance from medications and abnormal biochemistry [67]. The overall MARD of contemporary CGM sensors compared with reference glucose values from inpatient accuracy studies ranges between 9.8% and17.7% [68]. However, interpretation of these results requires consideration of the patient group studied, the number of matched paired glucose samples and the glucose levels evaluated during the study (i.e. hypo- and hyperglycaemia). Analysis of the accuracy of the Freestyle Libre 2 CGM system in 34 hospitalised adults on insulin therapy (342 matched paired CGM glucose and POC-BG samples) found an overall MARD of 17.7% [69]. However, the MARD during hyperglycaemia (>13.9 mmol/l) was 19.0% (34 matched glucose pairs). Although the MARD during hypoglycaemia (<3.9 mmol/l) was 7.6%, this was based on only five matched glucose pairs. An accuracy study involving 218 hospitalised participants (4067 matched glucose pairs) using a Dexcom G6 CGM system reported an overall MARD of 12.8% compared with POC-BG, with a higher MARD (18.8%, based on 52 matched pairs) in the hypoglycaemia range (2.8–3.9 mmol/l) [70].

Based on the above and other published evidence, several professional societies have produced clinical guidance and recommendations for CGM use in hospital settings [30, 71, 72]. Most still recommend confirmation of glucose levels using POC-BG monitoring because of uncertainty over inpatient sensor accuracy and performance as described above. The JBDS-IP (inpatient) guidelines also do not recommend CGM use for the management of hyperglycaemic emergencies, such as diabetic ketoacidosis. Most guidelines advise avoiding using CGM in specific inpatient situations where sensor performance may be affected, such as during periods of hypoperfusion or hypovolaemia, while on vasopressor therapy or while having MRI investigations [68]. Thus, further evidence on CGM accuracy, reliability and cost-effectiveness in hospitalised settings will be needed before CGM is accepted as standard of care (Table 2).

**Table 2:** Overview of and outlook for technological approaches for inpatient diabetes management

Examples	Benefits and limitations	Outlook
CDS	*Benefits* • Integration of glucose data with electronic insulin prescribing • At-risk patients or clinical areas can be flagged • Associated with a reduction in mean blood glucose and sliding scale use *Limitations* • Adherence limited by ease of access and user interface • Further improvements in user interface needed • Needs to be more accessible by healthcare providers	• Integrated with hospital glycaemic protocols • Insulin ordering and blood glucose readings linked
CGM	*Benefits* • Increased detection of hypoglycaemia, especially overnight *Limitations* • Limited data for inpatient use; mainly intensive care settings • Limited data on accuracy and reliability in inpatients	• Further data needed to determine accuracy and reliability • Integrated with hospital EMRs
AID system	*Benefits* • Improved glycaemic outcomes (i.e. increased time spent within target range and reduced time above range; no increase in hypoglycaemia risk) • Autonomous glucose control can be achieved and maintained with reduced staff workload *Limitations* • Limited data on clinical and hospital LOS outcome • Limited evidence on use outside research settings and cost-effectiveness	• Larger efficacy studies including health economic evaluation in inpatient populations needed • Further assessment needed in various inpatient populations

Adapted with permission from Thabit and Hovorka [95]. © 2017 Diabetes UK

### Automated insulin delivery

The basic principle of an AID system, sometimes known as a ‘closed-loop system’ or ‘artificial pancreas’, is the autonomous delivery of insulin based on available CGM and insulin data. Thus, CGM, insulin pump and control algorithms form the backbone of AID systems, with the algorithms being the ‘brain’ of the system in calculating the amount of insulin to be delivered [73]. Numerous studies have validated the efficacy and effectiveness of AID in outpatient settings, both in well-designed randomised clinical trials and observational real-world settings [74].

The first AID system to receive regulatory approval was the Medtronic 670G in 2017 and, since then, several more AID systems have been approved for clinical use. These systems, however, are only approved for outpatient use, and use a hybrid approach that relies on meal announcements to partially mitigate the pharmacokinetic and pharmacodynamic limitations of s.c. rapid-acting insulin [75]. An automated system delivering insulin in a glucose-responsive fashion would potentially address the unmet need for an effective and safe therapeutic approach for inpatient glucose management, while helping to reduce staff workload.

RCT data on AID in hospitalised individuals remain limited, with the majority of RCTs performed using the CamAPS HX AID system [76–78] (Table 1). Unlike other AID systems, the CamAPS HX is a fully closed-loop system requiring no meal announcement or bolus. This approach was adopted as a strategy to minimise healthcare professional and patient input, thereby potentially reducing the workload. In the largest RCT to date involving 136 participants with type 2 diabetes, use of a fully closed-loop system achieved significantly more time spent between 5.6 and 10.0 mmol/l glucose compared with conventional s.c. insulin therapy (65.8 ± 16.8% vs 41.5 ± 16.9%, *p*<0.001), without increasing the risk of hypoglycaemia [77]. The same system was tested in another RCT involving participants receiving enteral or parenteral nutrition [78]. Diabetes management in this cohort is known to be particularly challenging, requiring high levels of vigilance and workload from ward staff because of the carbohydrate content of nutritional support and unanticipated dislodgement of feeding tubes or discontinuation of feeding. In this study, time spent between 5.6 and 10.0 mmol/l glucose was significantly improved by AID compared with conventional s.c. insulin therapy (68.4 ± 15.5% vs 36.4 ± 26.6%; *p*<0.0001) [78].

More recently, a small non-randomised study (overall recruited *n*=22) tested the feasibility of the hybrid closed-loop Omnipod 5 AID, which uses a patch pump [79]. Trained nurses provided insulin boluses via the pump during meals in the study. The authors reported an overall time in range (3.9–10.0 mmol/l glucose) of 68 ± 16% in those with >48 h of CGM data, with a low incidence of hypoglycaemia. Future studies are being planned based on this promising start. Longer studies are still needed to evaluate clinical outcomes and cost-effectiveness to support AID use as standard of care in hospitalised individuals (Table 2).

## Implementing technology: barriers and steps toward clinical readiness

There has been an increased focus on improving inpatient care for people with diabetes over the past 5 years. This is seen through national initiatives such as Getting it Right First Time (GIRFT) in England [32], and the electronic clinical quality measures (eCQMS), which are part of the Hospital Inpatient Quality Reporting Program looking at measures of dysglycaemia in the USA [80]. There is limited but growing evidence that wider use of technology may benefit inpatient diabetes care and outcomes, but adoption of the latest diabetes-related technology for inpatient use can be hampered by a perceived increase in nursing and medical workload, concerns related to safety, and costs or lack of insurance reimbursement [81]. Therefore, in preparation for the wider use of technology, a change in practice is needed to overcome current barriers and ensure the seamless integration of this technology into usual clinical practice in the future. In the following sections, we outline key points related to supporting the implementation of technology in inpatient diabetes care.

### Education and support

The reality is that most people with diabetes receive their inpatient care not in tertiary or academic hospitals, where most of the aforementioned clinical studies were performed, but in smaller community or district hospitals where access to inpatient diabetes specialist teams is variable [82]. Thus, improving access to inpatient diabetes specialist teams should be prioritised to provide support, either physically or virtually, depending on local service needs. The COVID-19 global pandemic brought with it the realisation that the use of information technology and telehealth could help widen access to remote specialist advice and guidance, and virtual glucose management by inpatient diabetes teams has been shown to improve glycaemic outcomes [83].

Inpatient diabetes care is an evolving speciality that is currently undergoing the process of accreditation and certification [84], and guidance on the use of inpatient diabetes technology should form part of the assessment. Educating and training of ward teams on technology to improve diabetes care and achieve best practice is a potential role of inpatient diabetes teams. Educational materials and guidance for managing diabetes devices in hospitalised individuals have been published and can be adopted by inpatient teams [85]. As more evidence becomes available from clinical studies, and as technologies continue to advance, updated guidance will be necessary and should be integrated into the future education and training of inpatient teams.

In addition to healthcare professional-directed approaches, patient-directed self-management and use of wearable technologies during inpatient stays should also be supported when feasible. Individuals should be screened on admission to ensure that ward staff are aware and recognise if a person with diabetes is admitted to hospital with a CGM or insulin pump device. Most guidelines state that individuals who are not incapacitated, not in a diabetes emergency and able to self-manage their usual therapy should be allowed to continue using their CGM, pump or AID in hospital, as ward staff are unlikely to have expertise in their use. We have highlighted three real-life clinical scenarios (see Cases 1–3) in which use of diabetes technology in hospital and on discharge was supported.


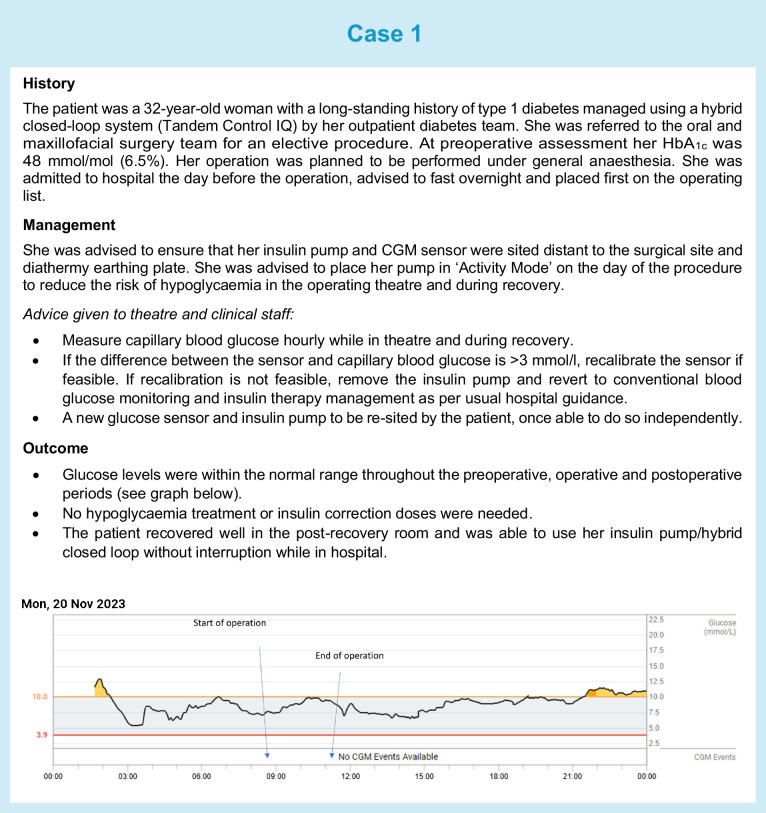



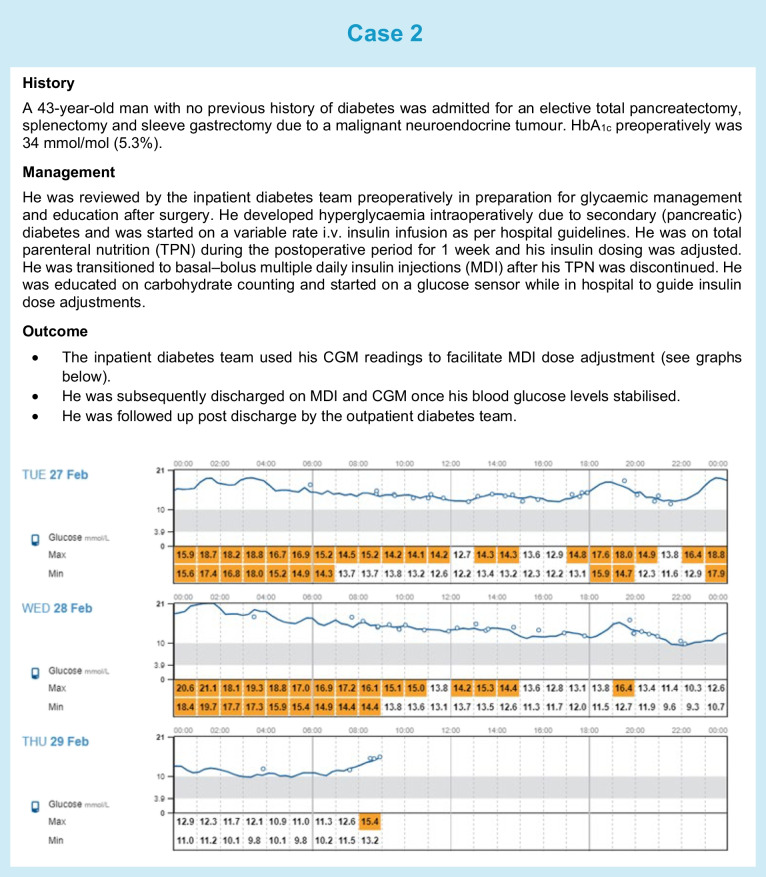



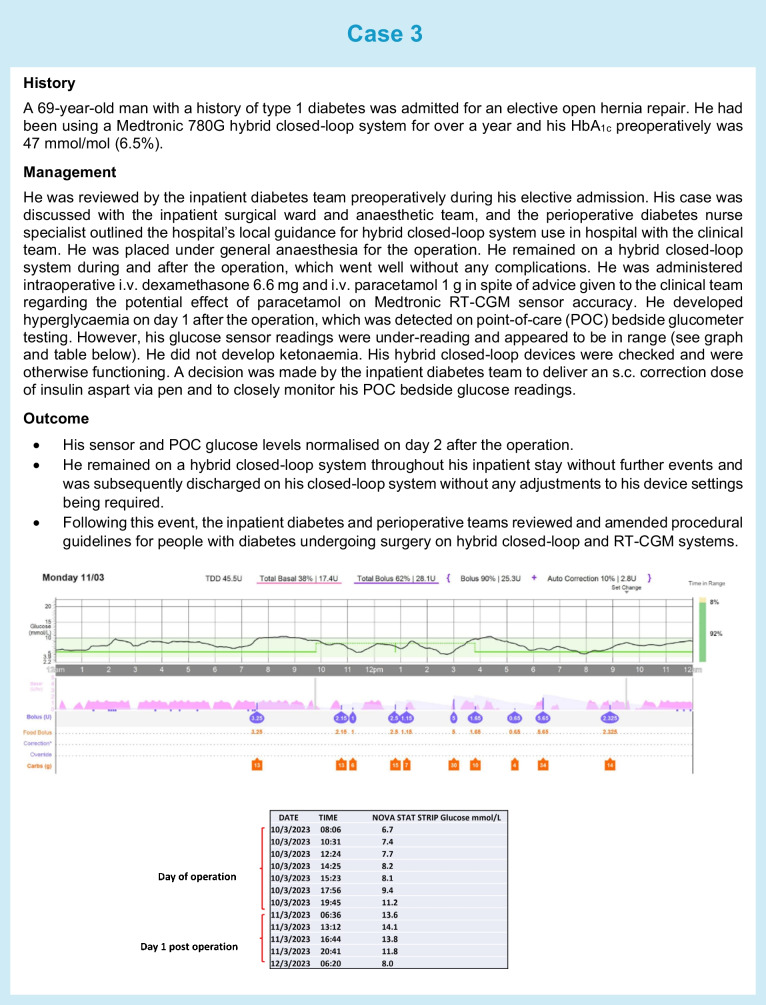


Currently, CGM and insulin pump device manufacturers provide user advice lines and technical support for their devices, including out of hours, and immediate replacement for faulty devices or consumables. Technical support from system manufacturers should also be readily accessible to inpatient teams for any future inpatient technologies in case of technical faults or emergency failures, to ensure safety.

### Seamless integration with workflow and EMRs

Technology attributes that support usability and adherence by ward staff are those that facilitate seamless integration with usual clinical workflows. A workflow is defined as ‘the set of tasks—grouped chronologically into processes—and the set of people or resources needed for those tasks that are necessary to accomplish a given goal’ [86]. Evidence suggests that, unless a new technological approach or system is aligned with the current clinical workflow and daily practice, technological adoption and quality of care may be adversely affected because of ‘workarounds’ [87].

At present, implementing CGM as standard of care in hospitalised individuals is complicated by the need to use different software applications to access data. This introduces burden, as healthcare staff need to electronically sign in to various platforms to view CGM data from different devices, causing interruption to their workflow. In addition, there may also be issues with accessing these platforms because of rigid hospital information technology and firewall systems. CGM data integration with EMRs is still limited, necessitating manual transcription of glucose values by ward staff when needing to document or act on glycaemic data. This barrier is also present when trying to access insulin delivery information from insulin pumps or AID systems in hospital.

Allowing hospital IT systems and EMRs to link with third-party applications, specifically those related to diabetes devices, has been suggested as a pragmatic approach to mitigate this barrier to diabetes technology adoption in hospitals [88]. On this basis, the Integration of Continuous Glucose Monitor Data in the Electronic Health Record (iCoDE) project was created with the intention to develop data standards and implementation policies to integrate CGM and other diabetes health technology data into EMRs. It includes key multinational and multiagency stakeholders and experts in the presentation, coding, regulation, analysis and clinical use of diabetes health technologies [89]. Discussions between industry, healthcare organisations and regulatory bodies are ongoing and still needed to address the technical and regulatory gaps surrounding data conformity and security standards of these technologies before wide implementation to ensure that patient rights and confidentiality are protected.

### Cost-effectiveness and economic evaluation

The spending on healthcare technology per capita across Europe has remained variable between countries over the past 20 years [90, 91]. Technology adoption in large healthcare organisations such as the UK National Health Service (NHS) relies on various set-up processes including implementation decisions and procurement, which themselves are influenced by factors such as complexity, scale and costs [92].

Because of the upfront and maintenance costs of equipment, technological competencies and staff training, healthcare providers may be disincentivised to adopt novel technologies in inpatient diabetes care without reliable evidence of cost-effectiveness and mechanisms for reimbursement. As an example, although adoption of CDS integrated with EMRs could enable workflow efficiency and cost savings over the longer term, it may be perceived to be financially risky by healthcare payers because of the short-term upfront cost required to purchase, install and train clinical staff on its use.

Health economic analysis of diabetes technology in the outpatient setting shows that, although short-term costs are increased, the long-term health gains and cost savings justify their use [93, 94]. The impact of shorter durations of technology use in hospitalised individuals, however, is yet to be determined. Given the initial high expenditure and potential decrease in clinical activity during training that may need to be accounted for when initiating these technologies, evidence to identify those individuals in hospital who might benefit the most from diabetes technologies would help support healthcare providers to make informed choices regarding appropriate use and reimbursement of these technologies. Until then, the full potential benefits and cost savings associated with diabetes technology use in hospital remain to be realised.

## Conclusion

Inpatient diabetes management remains challenging despite available guidelines because of the increasing diabetes prevalence and staff workload burden. Diabetes technology is currently going through a state of growth and development, with robust evidence available from outpatient settings, and has the potential to address unmet needs in inpatient care by improving efficacy, efficiency and safety. However, the field of technology in inpatient diabetes management remains underserved and under-researched. Further clinical and health economic evidence is needed to support its adoption by healthcare providers and payers. Current and future planning to implement workforce training and changes in practice is an important consideration if the seamless integration of technology in hospital is to be realised.

## Abbreviations

AID: Automated insulin delivery
CDS: Clinical decision support
CGM: Continuous glucose monitoring
EMR: Electronic medical record
JBDS: Joint British Diabetes Societies
LOS: Length of stay
MARD: Mean absolute relative difference
POC-BG: Point-of-care capillary blood glucose monitoring
RT-CGM: Real-time continuous glucose monitoring
